# Generation of High-Quality African Swine Fever Virus Complete Genome from Field Samples by Next-Generation Sequencing

**DOI:** 10.3390/v16020312

**Published:** 2024-02-18

**Authors:** Chuan Shi, Qinghua Wang, Yutian Liu, Shujuan Wang, Yongqiang Zhang, Chunju Liu, Yongxin Hu, Dongxia Zheng, Chengyou Sun, Fangfang Song, Xiaojing Yu, Yunling Zhao, Jingyue Bao, Zhiliang Wang

**Affiliations:** 1 China Animal Health and Epidemiology Center, Qingdao 266032, Chinaliuyutian@cahec.cn (Y.L.); liuchunju@cahec.cn (C.L.); zhengdongxia@cahec.cn (D.Z.); sunchengyou@cahec.cn (C.S.);; 2 BGI-Qingdao, BGI-Shenzhen, Qingdao 266555, China; 3 College of Life Sciences, University of Chinese Academy of Sciences, Beijing 518083, China

**Keywords:** African swine fever virus, next-generation sequencing, genome characterization, clinical samples

## Abstract

African swine fever (ASF) is a lethal contagious viral disease of domestic pigs and wild boars caused by the African swine fever virus (ASFV). The pandemic spread of ASF has caused severe effects on the global pig industry. Whole-genome sequencing provides crucial information for virus strain characterization, epidemiology analysis and vaccine development. Here, we evaluated the performance of next-generation sequencing (NGS) in generating ASFV genome sequences from clinical samples. Thirty-four ASFV-positive field samples including spleen, lymph node, lung, liver and blood with a range of Ct values from 14.73 to 25.95 were sequenced. For different tissue samples collected from the same sick pigs, the proportion of ASFV reads obtained from the spleen samples was 3.69–9.86 times higher than other tissues. For the high-viral-load spleen samples (Ct < 20), a minimum of a 99.8% breadth of ≥10× coverage was revealed for all the samples. For the spleen samples with Ct ≥ 20, 6/12 samples had a minimum of a 99.8% breadth of ≥10× coverage. A high average depth of sequencing coverage was also achieved from the blood samples. According to our results, high-quality ASFV whole-genome sequences could be obtained from the spleen or blood samples with Ct < 20. The high-quality ASFV genome sequence generated in this study was further used for the high-resolution phylogenetic analysis of the ASFV genomes in the early stage of the ASF epidemic in China. Our study demonstrates that NGS may act as a useful tool for efficient ASFV genome characterization, providing valuable information for disease control.

## 1. Introduction

African swine fever (ASF) is a lethal contagious viral disease of domestic pigs and wild boars caused by the African swine fever virus (ASFV). ASF affects African wild suids (warthogs and bushpigs) in an asymptomatic carrier state. Soft ticks serve as natural reservoirs and transmit the disease to suids. Since its first identification in Kenya in 1921, ASF has been reported in most countries in Africa, Europe, and Asia. It has also recently been introduced into Oceania (Timor-Leste and Papua New Guinea) and America (Dominica and Haiti) [1]. Over 50 countries are now affected by ASF, causing the death or culling of around ten million domestic pigs from 2016 to 2023 [2]. With no efficient vaccine or treatment available, the pandemic spread of ASF has seriously affected the global pig industry. 

ASFV is a member of the family *Asfarviridae*, belonging to nucleocytoplasmic large DNA viruses (NCLDVs). The viral particle of ASFV possesses an icosahedral morphology and a multilayered structure, with a diameter of 200–300 nm [3,4,5]. The genome of ASFV is a linear double-stranded DNA (dsDNA) molecule of 171 kb to 193 kb with terminal inverted repeats and hairpin loops at both ends [6,7]. The ASFV genome has a 125 kb conserved central region (CCR) flanked by the left and right variable regions. The CCR region contains genes involving virus replication, assembly, and host cell function modulation. The left and right variable regions contain multigene family (MGF) genes, including MGF 100, 110, 300, 360 and 505/530. Twenty-four genotypes of ASFV have been identified by a phylogenetic analysis based on the 440 bp length partial sequence of the B646L gene, which encodes the major structural protein p72 [8,9,10,11]. All 24 different genotypes of ASFV were found to be endemic in Africa. The ASFV strains currently circulating in Europe, Asia, Oceania and America belong to genotype II [12,13,14,15]. The ASFV genotype I and recombinant genotype I/genotype II ASFV strains were also recently reported in China [16,17]. 

The development of next-generation sequencing (NGS) and the reduction in the genome sequencing cost has enabled large-scale genome sequencing for ASFV. The genome characterization of circulating ASFV strains has expanded our knowledge of the genetic diversity and evolution of ASFV, providing valuable information for epidemiology studies and vaccine development. The whole-genome sequencing of ASFV has been widely used for virus strain characterization. Over 200 ASFV genome sequences of different origins and variable virulence have been determined and deposited in GenBank [7,18,19,20]. Different types of ASFV genome variation have been identified including the gain or loss of MGF members, deletion or insertion of genomic fragments, variation in the numbers of repeat units in the tandem repeat sequences, polymer tract variation and single nucleotide variation. Molecular epidemiology studies based on genome sequences have been conducted to reconstruct the evolution of ASFV globally or trace the origin and evolution of genotype I ASFV in Sardinia [21,22,23,24]. Genomic epidemiology analysis has demonstrated that genotype II ASFV in Eurasia has evolved into genetic clusters defined by eight anchor mutations [25]. The study also verified a strong monophyletic relationship among all the ASFV genome sequences from China and other Asian countries, which share a common ancestor with those from Central or Eastern Europe. The genomic surveillance of ASFV on the whole-genome level has also been used to monitor the ASFV variants on the rise in Germany [26]. In addition, the whole-genome sequencing of ASFV has contributed to vaccine candidate development [27,28]. 

Different methods have been developed for ASFV genome sequencing. Amplicon-based whole-genome sequencing uses many pairs of overlapping primers to amplify ASFV viral genome before sequencing [16]. However, amplicon failure may occur by mismatches between the targeted sequences and the primers, resulting from the recombination or large-fragment deletion in the ASFV genome [17,29,30]. The hybrid capture-based method uses predesigned biotinylated RNA baits for the specific capture enrichment of the ASFV genome [31]. The application of this method is limited due to the high cost of RNA baits. Next-generation sequencing (NGS) is currently the method routinely used for ASFV genome sequencing, having the advantages of a low cost and high accuracy [7,23,32]. One major challenge of NGS is the low abundance of the virus DNA present in primary clinical specimens compared to the overwhelming amount of host DNA. It is thus very important to improve the efficiency of NGS in ASFV genome sequencing from clinical samples. 

Here, we evaluated the performance of NGS in generating ASFV genome sequences in different kinds of clinical samples to choose the most efficient sample type. We also assessed the efficiency of NGS in low-ASFV-viral-load samples. The high-quality ASFV genome sequence generated in this study was used for the phylogenetic analysis of ASFV genomes in the early stage of the ASF epidemic in China. Our study provides valuable information for large-scale ASFV genome sequencing. It also highlights the important role of whole-genome sequencing in tracing the evolution of ASFV in Asia. 

## 2. Materials and Methods

### 2.1. Field Samples 

Thirty-four field samples from 27 infected pigs from an outbreak of ASF in Liaoning province in China on 16 October 2018 were selected for sequencing (Table 1). Each sample was tested with real-time PCR targeting the B646L gene. The cycle threshold (Ct) values for these samples ranged from 14.73 to 25.95. The samples were divided into three groups. Group 1 included 7 pairs of tissue samples, each pair comprising one low-Ct-value spleen sample (Ct value < 20) and another tissue sample (lymph node, lung or liver) from the same infected pig. Group 2 contained 12 spleen samples with Ct value ≥ 20. Group 3 contained 8 blood samples in which Ct values ranged between 16.84 and 25.95. Spleen and blood samples from the same individual pig were not compared because of the limitation of field samples we received.

### 2.2. Genome Sequencing

A total of 100 mg of tissue sample was homogenized and centrifuged. A total of 200 μL of tissue homogenate or blood was used for viral DNA extraction on a Kingfisher Flex system (Thermo Fisher Scientific, Waltham, MA, USA). The sequencing library was constructed following the manufacturer’s instructions for the MGIEasy-Fast Enzymatic DNA Library Preparation Kit (MGI Tech, Shenzhen, China). In brief, the extracted DNA was fragmented into 300 to 700 bp by Fast FS enzyme. The universal dual-index barcoding adapters were ligated to both ends of the DNA fragments. The fragments were further amplified using the UDB PCR primer mix. The resulting PCR products were purified and subjected to circularization. The single-strand circular DNA library was obtained using the MGIEasy-DNBSEQ One-step DNB Library Preparation Kit (MGI Tech, Shenzhen, China). Sequencing was performed according to the MGISEQ-2000RS protocol employing the PE150 mode to produce the raw paired-end reads of 150 bp. For the sequencing, 10 different libraries were pooled and simultaneously sequenced in one lane on an MGISEQ-2000RS sequencer. Each lane allows for the production of up to 100 gigabases. The total number of reads and the quality of sequences obtained from each library varies according to several factors such as extent of DNA degradation in sample, quality of library, pooling deviation, sequencing chemistry and sequencing bias.

### 2.3. Sequencing Data Analysis

Raw reads were cleaned by filtering the low-quality reads using SOAPnuke [33]. Host reads were removed by using BWA to map the reads to the swine reference sequence (GenBank accession no. GCF_000003025.6) [34]. Reference-based assembly of ASFV reads was performed by using MAQ to map the cleaned reads to the ASFV reference sequence China/LN/2018/1 (GenBank accession no. OP856591.1) [35]. The average depth of sequencing coverage was calculated as LN/G, where L is the read length, N is the number of reads and G is the genome length [36]. The breadth of coverage indicated the percentage of target bases that have been sequenced for a given number of times [37]. In accordance with our experience, a minimum of a 99.8% breadth of at least tenfold coverage of the whole ASFV genome was required to achieve a complete and accurate full-length ASFV genome sequence.

### 2.4. Genome Sequences Alignment and Phylogenetic Analysis

A total of 39 genotype II ASFV genomes collected from 15 countries from 2007 to 2020 described in a curated dataset were used for phylogenetic analysis [25]. Multiple nucleotide alignment of genome sequences was conducted using MAFFT software (version 7.475) [38]. Maximum likelihood (ML) phylogenetic trees were estimated by RAxML v8.2.12 using GTR + G nucleotide substitution model [39]. ML bootstrapping was performed with 1000 resamples to assess the robustness of tree topologies. The final tree was midpoint rooted by FigTree v1.4.2. (http://tree.bio.ed.ac.uk/software/figtree/ (accessed on 1 September 2022)). Mutations of single nucleotide variations (SNVs) in the coding region sequences of ASFV were used for phylogenetic network analysis with the Network software (version 10.2) (https://www.fluxus-engineering.com/ (accessed on 1 September 2022)) [40]. The data were run by the median joining network algorithm and the Steiner tree algorithm, respectively, with the epsilon parameter set to zero. 

## 3. Results

### 3.1. Performance of mNGS on Different Types of Tissue Samples

Seven pigs were used to investigate the performance of the NGS on different tissue samples. From each pig, both the spleen and another tissue sample, including lymph nodes, liver or lung, were used for the ASFV genome sequencing. As shown in Table 2, the Ct value difference between the spleen and the other tissue sample (−1.33 ± 1.59) collected from the same pig was insignificant (*p* = 0.07). 

From pig P01, 19 million reads were obtained for the spleen (Ct value 15.65) and lymph node (15.99), respectively. From the spleen sample, 307, 725 reads were identified as being ASFV-specific, representing 1.59% of the total reads. An average depth of 259× was achieved, producing a high-quality full-length genome sequence with a 100% breadth of ≥10× coverage. From the lymph node sample, only 85, 603 ASFV reads were obtained (0.43% of the total reads), making a genome sequence with 75× average depth and a 99.96% breadth of ≥10× coverage. The number of ASFV reads, the proportion of ASFV reads or the average depth of the ASFV genome in the spleen sample is approximately 3.59, 3.67 and 3.45 times that in the lymph node sample, respectively. In pig P02, P03 and P04, similar Ct values were found in the spleen and the other tissue sample. 

As shown in Table 2, when roughly the same number of total reads were obtained from the spleen and the other tissue sample, the number of the ASFV reads (*p* = 0.04), the proportion of the ASFV reads (*p* = 0.07) and the average depth of the ASFV genome (*p* = 0.03) obtained in the spleen samples was significantly higher than that in the other tissue samples. It was demonstrated that the spleen was more efficient for the ASFV genome sequencing than the other tissues, providing a high yield of ASFV reads for high-quality genome assembling with a high average depth of sequencing coverage.

**Figure 1:**
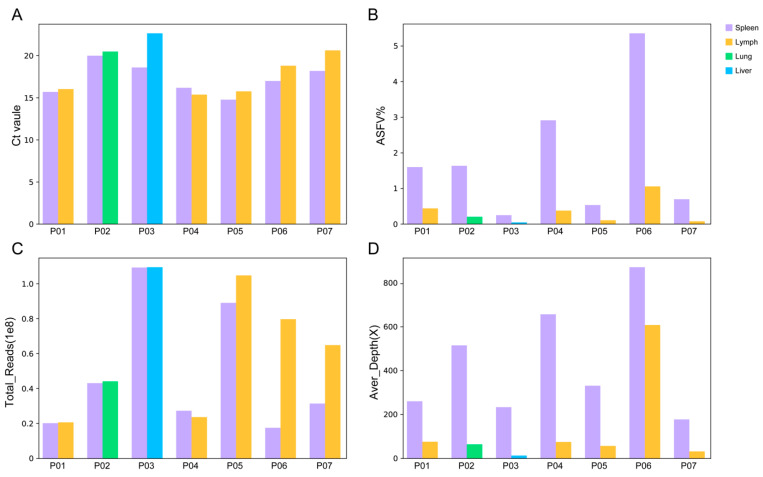
Performance of NGS on different types of tissue samples. (**A**) Ct value in different types of tissue samples used in this study. (**B**) The ratio of ASFV reads obtained by NGS in different types of tissue samples. (**C**) Total reads obtained in different tissue samples. (**D**) The average sequencing depth of the ASFV genome sequence in different tissue samples.

The influence of the sequencing quantity on the efficiency of NGS was further investigated. In pig P05, P06 and P07, the total reads obtained in the lymph node samples were 1.18, 4.59 and 2.07 times that obtained in the spleen samples. However, the proportion of ASFV reads in the spleen was 5.30, 5.09 and 9.86 times to that in the lymph node sample, producing a larger amount of ASFV reads and a higher average depth (Figure 1). In the lymph node samples of P05 and P07, although more than 60 million reads were achieved, the average depths were less than 60×, not reaching the required 99.8% breadth of ≥10× coverage. Further research needs to be conducted to determine the minimum required sequencing quantity for NGS-based ASFV genome sequencing. 

### 3.2. Performance of mNGS on Spleen Samples with Different Viral Loads

**Figure 2:**
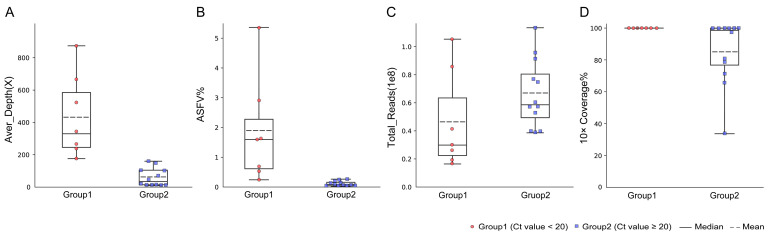
Performance of NGS on spleen samples with different viral loads. (**A**) Average sequencing depth of the ASFV genome sequence obtained in spleen samples with different Ct values. (**B**) The ratio of ASFV reads obtained by NGS in spleen samples with different Ct values. (**C**) Total reads obtained in spleen samples with different Ct values. (**D**) The ratio of breadth of ≥10× coverage in spleen samples with different Ct values.

To evaluate the efficiency of NGS on the low-viral-load samples, the spleen samples from 12 pigs with high Ct values were used for genome sequencing and compared with the spleen samples in group 1 (Table 3). For seven spleen samples with Ct < 20 in group 1 (Ct value from 14.73 to 19.95), the proportion of the ASFV reads ranged from 0.24% to 5.35% (1.85% on average). When 16 to 105 million total reads were sequenced for these samples, a range of 176× to 871× the average depth of the ASFV genome was achieved. It was revealed that the high-quality ASFV genome sequences with a 99.9% breadth of ≥10× coverage were obtained for all seven samples (Figure 2). 

For the 12 spleen samples with Ct ≥ 20 (Ct value from 20.04 to 24.28), the proportion of ASFV reads ranged from 0.02% to 0.26%. A range of 9× to 160× the average depth of the ASFV genome was achieved when 38 to 113 million total reads were sequenced. A total of 6 of the 12 samples had a high-quality ASFV genome with a 99.8% breadth of ≥10× coverage. In conclusion, the efficiency of NGS in generating a high-quality complete ASFV genome decreased dramatically in the spleen samples with a low viral load. 

### 3.3. Performance of mNGS on Blood Samples

**Figure 3:**
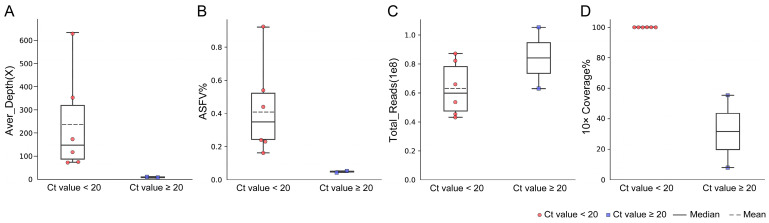
Performance of NGS on blood samples. (**A**) Average sequencing depth of the ASFV genome sequence obtained in blood samples with different Ct values. (**B**) Theproportion of ASFV reads obtained by NGS in blood samples with different Ct values. (**C**) Total reads obtained in blood samples with different Ct values. (**D**) The ratio of breadth of ≥10× coverage in blood samples with different Ct values.

The blood samples from eight pigs with Ct values from 16.84 to 25.95 were used to evaluate the efficiency of NGS (Table 4). For six blood samples with Ct < 20 (Ct value ranging from 16.84 to 18.97), the proportion of the ASFV reads ranged from 0.15% to 0.92% (0.42% on average). When sequencing 43 to 87 million total reads, a range of 72× to 629× the sequencing depth of the ASFV genome was produced. It was revealed that a 99.9% breadth of ≥10× coverage was achieved for all six samples (Figure 3). 

For two blood samples with Ct values of 21.40 and 25.95, the ratio of the ASFV reads was found to be 0.03% and 0.04% when sequencing for 63 and 105 million total reads. From these low-viral-load blood samples, 11× and 5× the sequencing depth of the ASFV genome were achieved, resulting in a 54.03% and 8.8% breadth of ≥10× coverage, respectively. According to our results, a high-quality complete ASFV genome could be obtained in blood samples with Ct < 20. 

### 3.4. Performance of mNGS for the Detection of SNVs

The complete ASFV genome sequences generated in this study were further compared to define the accuracy of the NGS. Eleven samples not reaching the required 99.8% breadth of ≥10× coverage were excluded from the analysis, including three samples (P03 Liver, P05 Lymph node, and P07 Lymph node) in group 1, one sample in group 2 (P12 Spleen), five samples in group 3 (P22–P26 Spleen), and two samples in group 4 (P26 Blood and P27 Blood). For the other 23 samples from 19 individual pigs, the full-length ASFV genome sequences were obtained and further compared. 

These genome sequences were aligned and compared with the reference genome China/LN/2018/1. It was revealed that all the ASFV genomes obtained in this study were identical. The final assembly of the ASFV China/LN/2018/2 genome obtained from the spleen sample of pig P06 with the highest sequencing depth (871 reads per nt) was used for further analysis. As shown in Table 5, the comparison of the China/LN/2018/2 genome with the reference genome China/LN/2018/1 revealed one non-synonymous single nucleotide variation (SNV) at C9042T (G260A/R87Q in CDS of MGF110-4L) and one single nucleotide insertion at nucleotide position 21,794 in the intergenic region between MGF 300-1L and MGF 300-2R. Variations in two highly variable poly G or poly C tracts were also found: one located at site 1382 (poly C_10_ in China/LN/2018/1 and poly C_8_ in China/LN/2018/2) in the 5′ untranslated region of MGF 360-1La, the other located at site 14,225 (poly C_13_ in China/LN/2018/1 and poly C_11_ in China/LN/2018/2) which caused the truncation of MGF 110-14L from a length of 272 amino acids to 116 amino acids.

### 3.5. Phylogenetic Analysis

**Figure 4:**
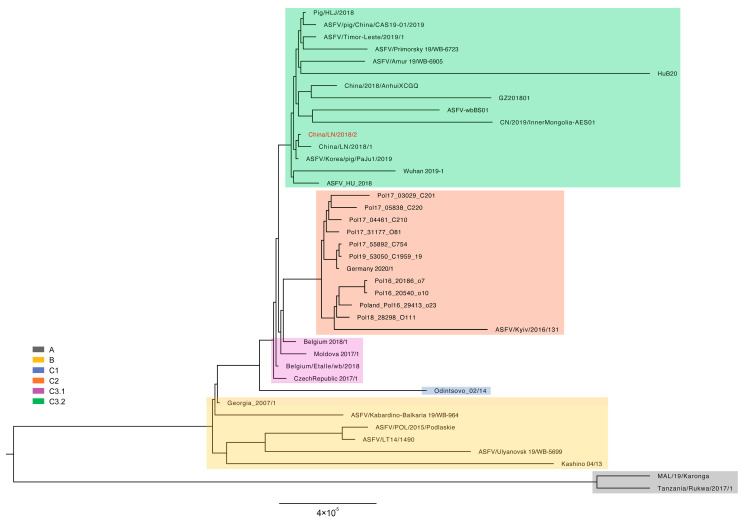
Maximum likelihood phylogenetic tree of genotype II ASFV genome sequences. The tree is midpoint rooted. The scale bar is given in the numbers of substitutions per site. The posterior probability higher than 0.7 are shown at the corresponding nodes. Cluster assignment is shown by color: grey for clade A, yellow for clade B, blue for clade C1, orange for clade C2, purple for clade C3.1 and green for clade C3.2.

The ASFV China/LN/2018/2 genome was added to other genotype II ASFV genomes in the curated dataset as previously reported, making a total of 40 ASFV genome sequences for further analysis. The maximum likelihood (ML) phylogenetic dendrogram of ASFV was constructed. The ML tree showed ASFV China/LN/2018/2 was grouped into cluster C3.2, with 10 strains collected from China from 2018 to 2020 (Figure 4). As expected, the ASFV China/LN/2018/2 was most closely related to ASFV China/LN/2018/1, a strain collected from the first outbreak of ASF in Shenyang, Liaoning, China. The tree topology indicated that high-quality ASFV genome sequencing enabled high-resolution phylogenetic analysis to trace the evolution of ASFV strains during an epidemic. 

**Figure 5:**
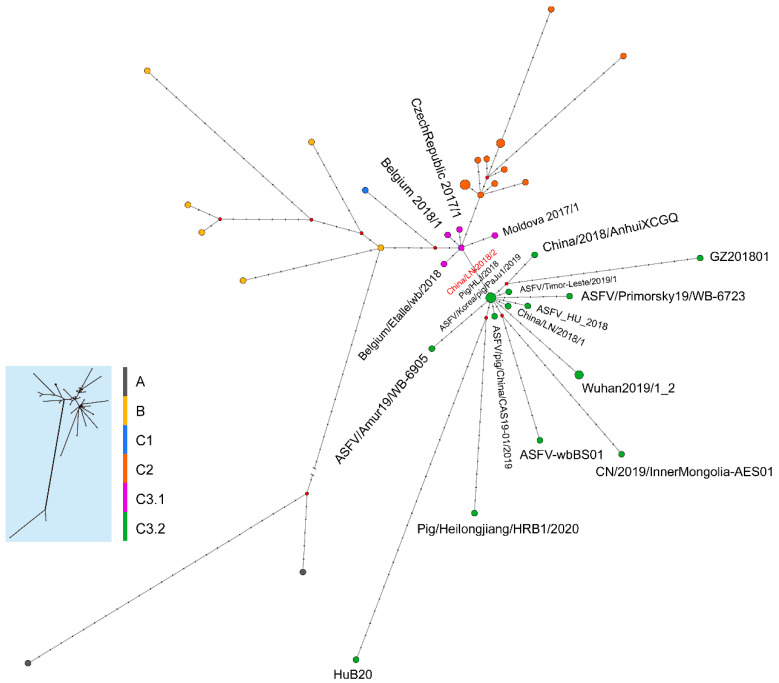
Phylogenetic network of genotype II ASFV genomes. The network was constructed from the SNVs in the coding region of genome sequences. Circle nodes represent ASFV strains, which are proportional to the number of taxa. Each notch on the links represents a mutation event. Red dots indicate putative ancestor nodes. Cluster assignment is shown by color. The median joining network algorithm and the Steiner algorithm were used.

The median joining phylogenetic network was constructed based on the SNVs to explore the evolutionary relationships between ASFV China/LN/2018/2 and other ASFV genotype II genomes in China. ASFV China/LN/2018/2 was clustered into the ancestral node in cluster C3.2 with one sequence from Heilongjiang province in China on 5 September 2018, and the other from South Korea on 16 September 2019 (Figure 5). 

## 4. Discussion

Here, we conducted an in-depth study on the performance of NGS in generating ASFV genome sequences from clinical samples. Our study demonstrated that the spleen was the most efficient sample for ASFV genome sequencing. The proportion of ASFV-specific reads obtained in the spleen was higher than that in the lymph node and other tissue samples. It has been widely demonstrated that the spleen is the main target organ of ASFV. In pigs that naturally died of experimental infection with virulent genotype II ASFV, the ASFV DNA quantity detected by p72-targeted real-time PCR in the spleens was significantly higher than in the other organs [41]. In another controlled experimental study, in biological samples collected in 82 pigs killed 5 days after the natural consumption of ASFV in feed and liquid, the probability of detecting ASFV by real-time PCR and virus isolation was spleen > lymph nodes > tonsil > serum > feces. All the biological sample types had higher ASFV detection rates by PCR when compared to virus isolation except for the spleen, where virus isolation had a higher diagnostic sensitivity. It was revealed that the ASFV detection rates by real-time PCR in the spleen (70.6%) were slightly higher than that in the lymph node (58.8%). However, the ASFV detection rates by virus isolation in the spleens (94.1%) were much higher than those in the lymph nodes (29.4%) [42]. Our study provides solid evidence for addressing the spleen as the sample of choice to obtain a high-quality ASFV genome sequence. Our results also revealed that the lymph nodes could be an alternative tissue sample type for ASFV genome sequencing in the case that a spleen sample was not available. The proportion of ASFV reads generated by NGS in the different samples in this study ranged from 0.02% to 5.35%, making it difficult to determine the cut-off of the total number of reads for generating a high-quality ASFV whole-genome sequence. More samples collected from different ASF outbreaks will be used to further investigate NGS’s performance in generating high-quality ASFV genome sequences, including the cut-off of the total number of reads.

Anticoagulated whole blood is the second choice of sample for obtaining high-quality ASFV genome sequences. Previous studies demonstrate that anticoagulated whole blood is the most sensitive sample for ASFV DNA detection. A high load of ASFV DNA was detected in blood in the early, clinical and later phases of ASFV experimental infection [43]. At 5~8 days post-infection, the ASFV DNA detected in the blood increased dramatically from 10^2^ copies/μL to 10^7^ copies/μL [42]. Whole blood samples are suitable for high-throughput ASFV genome sequencing, considering there is no need for homogenization. This fits for the passive surveillance of ASFV in pig-dense areas, especially in abattoirs. In this study, the efficiency of NGS on the spleen and blood samples from the same individual pig was not compared because of the limitation of field samples we received. Further study will be conducted to compare the efficiency of NGS on the spleen and blood samples. 

We did not see a strong correlation between the ASFV viral load determined by the qPCR and the proportion of ASFV reads in the genome sequencing data. There are several possible contributing factors to this, including the extent of DNA degradation during sample collection in the field and shipment to the laboratory, the content of other DNA sequences in the samples, the variable ASFV amplification before the sequencing step, particularly for the rolling circle amplification. A study on the detection of Torque teno virus in children with leukemia by metagenomic sequencing and by quantitative PCR indicated that this is not unique to our samples [44]. The performance of genome sequencing, qPCR and virus isolation needs to be further evaluated by using samples collected from a controlled experimental study. 

NGS produced high-quality full-length ASFV genome for samples with a high viral load but did not produce a whole ASFV genome sequence for the high-Ct-value samples in the present study. The efficiency of the ASFV genome sequencing decreased dramatically in the samples with a lower viral load (Ct ≥ 20). Several methods have been developed to enrich the viral DNA and improve the efficiency of ASFV genome sequencing. An effective workflow combining target enrichment, Illumina and Nanopore sequencing has been developed for ASFV whole-genome sequencing. By using the ASFV target enrichment protocol, the proportion of ASFV-specific reads increased from 0.05% to 83.89% in one pig spleen sample [32]. A novel approach for DNA enrichment, based on the separation of methylated and un-methylated DNA was evaluated in the sequencing of the ASFV genome in five pig blood samples and eight tick samples [45]. It was revealed that the enrichment of samples in un-methylated DNA for the sequencing of the ASFV genome represented extra work and cost without a significant improvement in the final results for the very-low-ASFV-load samples. A C18 spacer MDA (Multiple Displacement Amplification)-combined host DNA exhaustion strategy was used to remove the background DNA and sequence two uncultured ASFV positive samples. The ASFV reads had been raised 46 and 112 times, respectively [46]. The efficiency of these methods in generating high-quality ASFV genome sequences from low-viral-load clinical samples needs to be further investigated by using more reference and clinical samples. 

High-depth whole-genome sequencing is the ‘gold standard’ for ASFV variant identification because it can interrogate all variant types, including SNVs, indels, structural variants, and TRS copy number variation. The ability to detect variations, especially SNVs and small indels, in ASFV variants by NGS is reduced by the low base quality and by the non-uniformity of coverage. Increasing the sequencing depth can reduce the false discovery rate for variant calling. Human genome sequencing using Illumina short-read technology has suggested that an average mapped depth of 50× would be required to allow for the reliable calling of SNVs and small indels for >94% of the genome [47]. The requirements for the sequencing depth for highly accurate SNV calls from the ASFV genome need to be further investigated. 

The tree topology indicated that high-quality ASFV genome sequencing enabled a high-resolution phylogenetic analysis to trace the evolution of the ASFV strains during an epidemic. The phylogenetic analysis of the genomic sequences revealed that genotype II ASFV has evolved into different genetic clusters with temporal and spatial correlation since being introduced into Europe and Asia [25]. The genomic surveillance of ASFV on the whole-genome level has identified at least ten variants characterized by high-impact mutations, including SNPs, indels, TRSs, and insertions in Germany [26]. ASFV genomes harboring different types of variation, including SNPs, indels, TRSs, fragment insertions/deletions, and recombination, have been identified in China since its introduction in 2018 [17,30,48]. It is, therefore, very important to closely monitor the evolution of the ASFV genome through high-quality whole-genome sequencing. 

In present study, NGS was tested on a limited number of field samples. More samples collected from different outbreaks of ASF should be used to confirm the present evaluation in further and future investigation. The limit of detection of NGS in generating ASFV genome sequences should be evaluated using some reference viral stock as well as tissues or blood samples in our further study. 

## 5. Conclusions

In conclusion, we report the performance of NGS in generating high-quality ASFV genome sequences from clinical samples. According to our results, high-quality ASFV whole-genome sequences could be obtained from spleen or blood samples with Ct < 20. Our study demonstrates that NGS may be a useful tool for efficient ASFV genome characterization, providing valuable information for disease control. 

## Figures and Tables

**Table 1:** List of field samples used in this study.

Group	Pig	Type	Ct value	Location	Date
Group 1	P01	Spleen	15.65	Liaoning	16 October 2018
	P01	Lymph node	15.99	Liaoning	16 October 2018
	P02	Spleen	19.95	Liaoning	16 October 2018
	P02	Lung	20.44	Liaoning	16 October 2018
	P03	Spleen	18.55	Liaoning	16 October 20188
	P03	Liver	22.60	Liaoning	16 October 2018
	P04	Spleen	16.14	Liaoning	16 October 2018
	P04	Lymph node	15.33	Liaoning	16 October 2018
	P05	Spleen	14.73	Liaoning	16 October 2018
	P05	Lymph node	15.72	Liaoning	16 October 2018
	P06	Spleen	16.95	Liaoning	16 October 2018
	P06	Lymph node	18.76	Liaoning	16 October 2018
	P07	Spleen	18.14	Liaoning	16 October 2018
	P07	Lymph node	20.58	Liaoning	16 October 2018
Group 2	P08	Spleen	20.04	Liaoning	16 October 2018
	P09	Spleen	20.28	Liaoning	16 October 2018
	P10	Spleen	20.30	Liaoning	16 October 2018
	P11	Spleen	20.50	Liaoning	16 October 2018
	P12	Spleen	20.53	Liaoning	16 October 2018
	P13	Spleen	20.60	Liaoning	16 October 2018
	P14	Spleen	21.10	Liaoning	16 October 2018
	P15	Spleen	21.57	Liaoning	16 October 2018
	P16	Spleen	22.00	Liaoning	16 October 2018
	P17	Spleen	22.19	Liaoning	16 October 2018
	P18	Spleen	22.48	Liaoning	16 October 2018
	P19	Spleen	24.28	Liaoning	16 October 2018
Group 3	P20	Blood	16.84	Liaoning	16 October 2018
	P21	Blood	17.72	Liaoning	16 October 2018
	P22	Blood	18.04	Liaoning	16 October 2018
	P23	Blood	18.25	Liaoning	16 October 2018
	P24	Blood	18.35	Liaoning	16 October 2018
	P25	Blood	18.97	Liaoning	16 October 2018
	P26	Blood	21.40	Liaoning	16 October 2018
	P27	Blood	25.95	Liaoning	16 October 2018

**Table 2:** Performance of mNGS on different types of tissue samples.

Pig	Type	Ct	Total Reads	Swine Reads	Host%	ASFV Reads	ASFV%	Aver Depth (×)	≥10× Coverage
P01	Spleen	15.65	19,300,794	19,039,879	98.65%	307,725	1.59%	259	100%
P01	LN *	15.99	19,753,224	19,482,394	98.63%	85,603	0.43%	75	99.96%
P02	Spleen	19.95	41,434,017	40,796,077	98.46%	675,114	1.63%	514	100%
P02	Lung	20.44	42,428,273	42,135,799	99.31%	83,171	0.20%	63	99.91%
P03	Spleen	18.55	105,357,666	104,789,969	99.46%	254,528	0.24%	232	99.93%
P03	Liver	22.60	105,531,945	104,924,581	99.42%	42,239	0.04%	12	56.41%
P04	Spleen	16.14	26,182,449	25,587,744	97.73%	761,136	2.91%	656	100%
P04	LN	15.33	22,644,180	22,397,705	98.91%	84,216	0.37%	74	99.99%
P05	Spleen	14.73	85,820,966	85,264,227	99.35%	452,122	0.53%	330	99.95%
P05	LN	15.72	101,012,071	100,567,586	99.56%	100,290	0.10%	56	99.77%
P06	Spleen	16.95	16,732,552	16,047,605	95.91%	894,899	5.35%	871	100%
P06	LN	18.76	76,822,410	76,034,726	98.97%	802,947	1.05%	607	100%
P07	Spleen	18.14	30,133,820	29,862,047	99.10%	207,573	0.69%	176	99.98%
P07	LN	20.58	62,476,949	62,132,807	99.45%	43,865	0.07%	31	99.19%

* LN represents lymph node.

**Table 3:** Performance of NGS on spleen samples with different viral load.

Group	Pig	Type	Ct	Total Reads	Swine Reads	Host%	ASFV Reads	ASFV%	Aver Depth (×)	≥10× Coverage
Group 1	P01	Spleen	15.65	19,300,794	19,039,879	98.65%	307,725	1.59%	259	100%
	P02	Spleen	19.95	41,434,017	40,796,077	98.46%	675,114	1.63%	514	100%
	P03	Spleen	18.55	105,357,666	104,789,969	99.46%	254,528	0.24%	232	99.93%
	P04	Spleen	16.14	26,182,449	25,587,744	97.73%	761,136	2.91%	656	100%
	P05	Spleen	14.73	85,820,966	85,264,227	99.35%	452,122	0.53%	330	99.95%
	P06	Spleen	16.95	16,732,552	16,047,605	95.91%	894,899	5.35%	871	100%
	P07	Spleen	18.14	30,133,820	29,862,047	99.10%	207,573	0.69%	176	99.98%
Group 2	P08	Spleen	20.04	95,779,150	94,301,391	98.46%	123,268	0.13%	106	99.98%
	P09	Spleen	20.28	91,428,758	90,842,633	99.36%	223,638	0.24%	160	99.94%
	P10	Spleen	20.30	76,917,858	76,437,533	99.38%	202,494	0.26%	149	99.85%
	P11	Spleen	20.50	113,506,138	112,655,479	99.25%	98,136	0.09%	73	99.98%
	P12	Spleen	20.53	39,908,094	39,648,842	99.35%	15,519	0.04%	12	65.69%
	P13	Spleen	20.60	74,872,389	74,307,643	99.25%	150,682	0.20%	104	99.88%
	P14	Spleen	21.10	38,853,338	38,575,461	99.28%	53,950	0.14%	47	99.89%
	P15	Spleen	21.57	52,842,857	52,245,719	98.87%	20,270	0.04%	15	78.61%
	P16	Spleen	22.00	57,136,899	56,724,036	99.28%	24,979	0.04%	17	80.78%
	P17	Spleen	22.19	57,246,209	57,032,875	99.63%	38,330	0.07%	22	97.28%
	P18	Spleen	22.48	39,724,759	39,441,332	99.29%	16,451	0.04%	13	71.31%
	P19	Spleen	24.28	60,321,322	59,628,674	98.85%	12,357	0.02%	9	33.76%

**Table 4:** Performance of NGS on blood samples with different viral loads.

Pig	Type	Ct	Total Reads	Swine Reads	Host%	ASFV Reads	ASFV%	Aver Depth (×)	≥10× Coverage
P20	Blood	16.84	43,110,963	42,600,180	98.82%	191,101	0.44%	177	99.99%
P21	Blood	17.72	53,662,886	53,281,393	99.29%	79,969	0.15%	72	99.97%
P22	Blood	18.04	45,388,551	45,162,234	99.50%	106,663	0.23%	77	99.92%
P23	Blood	18.25	87,175,246	86,377,934	99.09%	805,588	0.92%	629	100%
P24	Blood	18.35	82,171,243	81,563,167	99.26%	441,883	0.54%	362	99.99%
P25	Blood	18.97	65,916,159	65,526,088	99.41%	150,578	0.23%	114	99.94%
P26	Blood	21.40	62,907,376	62,631,286	99.56%	18,247	0.03%	11	54.03%
P27	Blood	25.95	105,264,301	104,625,443	99.39%	43,435	0.04%	5	8.80%

**Table 5:** Variations between the China/LN/2018/2 genome and the reference genome China/LN/2018/1.

Site	China/LN/2018/1	China/LN/2018/2	Type	CDS	CDS Variation	AA Variation
1382	poly C_10_	poly C_8_	poly G/C	5′ UTR MGF 360-1La	/	/
9042	C	T	SNV	MGF110-4L	G260A	R87Q
14,225	poly C_13_	poly C_11_	poly G/C	MGF 110-14L	G346–, G347–	G116 * (Truncation)
21,794	-	C	Insert	IGR MGF 300-1L/2R	/	/

* indicates stop codon.

## Data Availability

The genome sequence data generated in this research have been deposited in the GenBank database under accession number OR958825.
